# External fixation is not superior to K-wire fixation in pediatric patients with high-level extension-type supracondylar humeral fractures

**DOI:** 10.3389/fsurg.2026.1853760

**Published:** 2026-07-15

**Authors:** Nan Yang, Yan Zhang, Hui Qin

**Affiliations:** Department of Orthopedics, Shanghai Sixth People’s Hospital, Shanghai Jiao Tong University School of Medicine, Shanghai, China

**Keywords:** Baumann angle, external fixation, K-wire fixation, range of motion, supracondylar humerus fracture

## Abstract

**Objectives:**

Supracondylar humerus fractures (SHFs) are among the most common fractures in pediatric patients. The following two surgical options are available for high-level extension-type pediatric supracondylar humeral fractures: Kirschner wire (K-wire) fixation and lateral external fixation. Evidence comparing the clinical outcomes of these two procedures remains insufficient. This study aimed to compare functional and radiological outcomes between the two surgical approaches.

**Methods:**

This retrospective comparative study included 52 pediatric patients (15 treated with external fixation and 37 with K-wire fixation) with a minimum follow-up of 1 year. Outcome measures included open reduction rate, post-operative carrying angle, Baumann angle, humeral-capitellar angle, and complication rates (infection, ulnar nerve injury, and unacceptable cubitus varus). Elbow range of motion (ROM) was evaluated at serial follow-up points.

**Results:**

No ulnar nerve injury, major loss of reduction, pin-tract infection, or elbow stiffness occurred in either group. The only significant between-group difference was a lower open reduction rate in the external fixation group (*P* = 0.042). No statistically significant differences were observed in Baumann angle, humeral-capitellar angle, Flynn grade, carrying angle, or elbow ROM between the groups.

**Conclusions:**

K-wire fixation and external fixation provide similarly satisfactory functional and radiological outcomes. External fixation offers no significant advantage over K-wire fixation for high-level extension-type supracondylar humeral fractures.

## Introduction

Supracondylar humerus fractures (SHFs) in children account for more than 50% of elbow fractures and represent the second most common pediatric fracture type, with an annual incidence of 308 per 100,000 children (1–3). An SHF is an extra-articular fracture; delayed or inadequate treatment may lead to elbow range of motion (ROM) limitation, malunion, neurovascular compromise, cubitus varus, or Volkmann's ischemia (4–7). SHFs are classified into extension (98%, distal fragment posteriorly displaced) and flexion types (2%, distal fragment anteriorly displaced) (4). The extension type is further graded by the Gartland classification as follows: Type I (non-displaced), Type II (posterior cortical hinge intact), and Type III (completely displaced fracture) (8).

Gartland Type I fractures are typically managed conservatively with cast immobilization. For Gartland Type II fractures, whether surgery is necessary remains controversial, as does the optimal surgical strategy for Gartland Type III fractures. Traditionally, Type II fractures are treated with closed reduction and percutaneous pinning (CRPP) using Kirschner wires (K-wires) and Type III fractures are treated with CRPP or open reduction and internal fixation (9, 10). In 2008, Slongo et al. introduced lateral external fixation for SHFs, with favorable outcomes (11). No consensus has yet been reached regarding the optimal treatment of a high-level SHF. Studies have suggested that external fixation is biomechanically more stable than crossed K-wires and may reduce open reduction rate, operative time, intraoperative fluoroscopy, and ulnar nerve injury (11–15). However, no consensus has been reached. This retrospective study compared the clinical and radiological outcomes of K-wire fixation versus external fixation for high-level extension-type SHFs in children.

## Methods

### Study design

A retrospective comparative study was conducted involving 52 pediatric patients with Gartland Type II or III extension-type SHFs. All the patients received standardized preoperative evaluations and perioperative management. Diagnosis was based on clinical examination and radiographs. Patients with open fractures, concomitant neurovascular injury, or combined injuries were excluded. The reduction criteria were as follows: Baumann angle <80° on anteroposterior radiograph and anterior humeral line passing through the capitellum on the lateral view (16). Both groups followed the same reduction criteria. Patients were allocated to K-wire fixation (Group 1) or external fixation (Group 2) based on the surgeon's discretion according to clinical practice and fracture characteristics. Patient data included age, gender, injured side, and injury-to-surgery interval. The study was approved by the Ethics Committee of Shanghai Sixth People's Hospital. Written informed consent was obtained from the legal guardians of all participants.

### Surgical procedure

**Figure 1:**
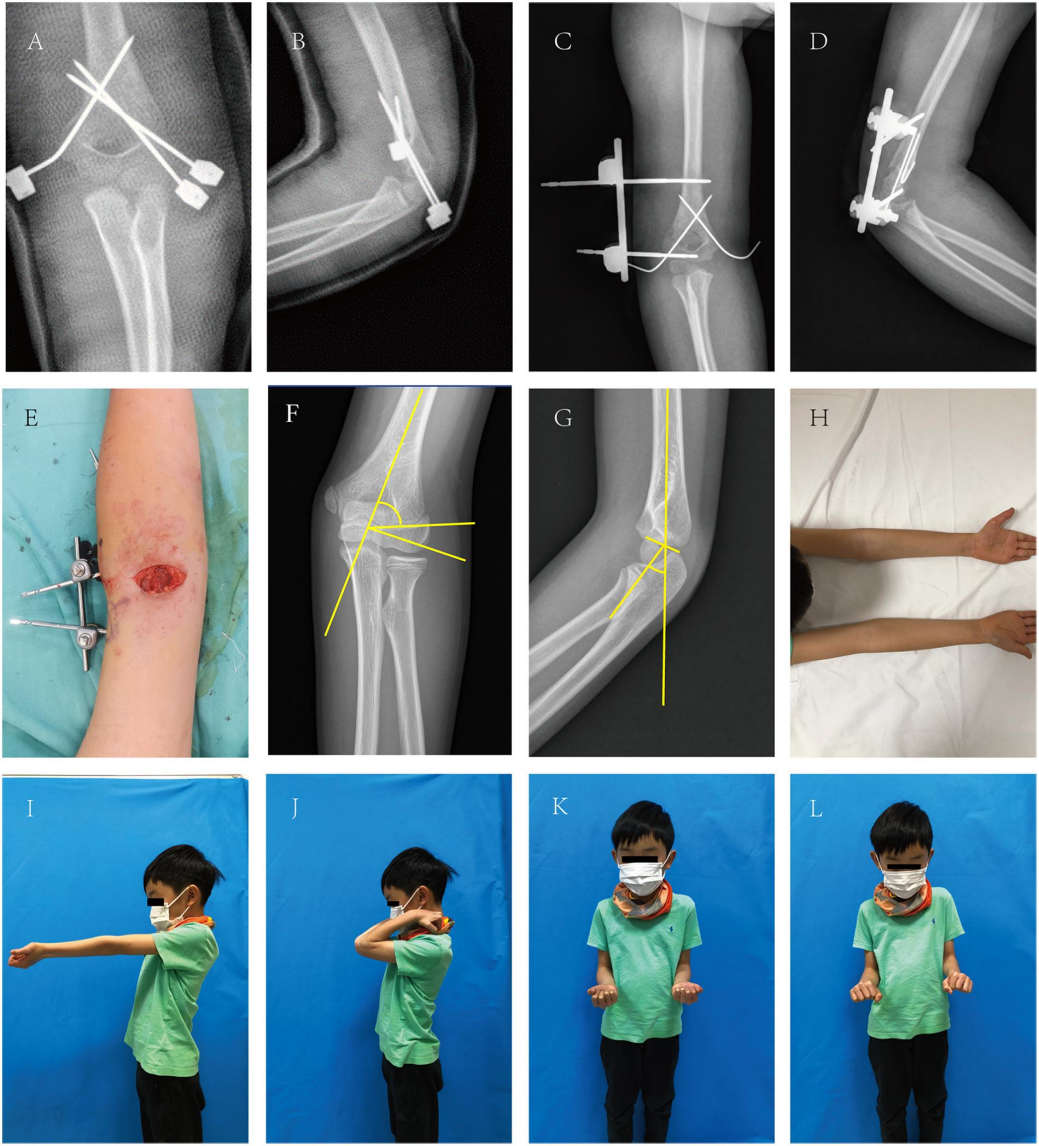
**(A,B)** Post-operative X-ray images of an SHF case treated with closed reduction, K-wire fixation, and a long arm cast. **(C,D)** External fixation. **(E)** The open reduction incision using the anterior approach. **(F)** Baumann angle. **(G)** Humeral-capitellar angle. **(H)** Carrying angle. **(I,J)** Assessment of elbow ROM. **(K,L)** Elbow rotational movement.

In Group 1 (K-wire fixation), surgery was performed with the patient supine under general anesthesia. Closed reduction was performed and confirmed under fluoroscopy. The fracture was stabilized with two lateral K-wires and one medial K-wire (2.0 mm). For patients in whom closed reduction had failed, open reduction was performed via a minimally invasive anterior approach through a small incision (Figure 1E). A transverse incision approximately 2–3 cm in length was made along the skin crease in the anterior cubital fossa. After incising the skin and subcutaneous tissue, the lateral antebrachial cutaneous nerve was identified and protected. The lateral border of the biceps brachii was bluntly dissected and medially retracted. The brachialis muscle was then exposed. A longitudinal incision was made along the midline of the brachialis muscle to directly reach the fracture site at the anterior aspect of the distal humerus. Any hematomas and interposed soft tissue were removed at the fracture ends (17). The fracture was reduced and fixed with two lateral and one medial 2.0 mm K-wires (16) (Figures 1A,B). Wounds were closed without drainage. Closed reduction cases were immobilized in a long arm cast at 90° elbow flexion. Open reduction cases were initially immobilized in a long posterior splint at 90° flexion during the swelling phase, then converted to a long arm cast at the same flexion angle after swelling subsided.

In Group 2 (external fixation), surgery was performed under general anesthesia. A 3-mm Schanz pin was inserted 3–5 cm proximal to the fracture line, avoiding the radial nerve. A second Schanz pin was placed in the distal fragment. These pins were used as joysticks to assist the closed reduction. Tube-to-tube clamps and a carbon fiber rod were loosely applied to connect the pins. Final reduction was verified under fluoroscopy; pins were inserted perpendicular to the long axis of each fracture fragment. For additional rotational stability, two 2.0 mm K-wires were inserted retrograde from the distal fragment across the fracture line into the proximal cortex (one medial, one lateral) (Figures 1C,D). In cases where closed reduction failed, open reduction was performed via the same minimally invasive anterior approach as in Group 1. Wounds were closed without drainage.

### Post-operative care and follow-up procedure

In Group 1, the K-wires and cast were removed simultaneously at 1 month post-operatively. Early active elbow ROM exercises were encouraged immediately after removal. In Group 2, the supplementary K-wires were removed at 1 month; the external fixator was removed at 2 months post-operatively. Active elbow ROM exercises were initiated after the fixator was removed.

**Figure 2:**
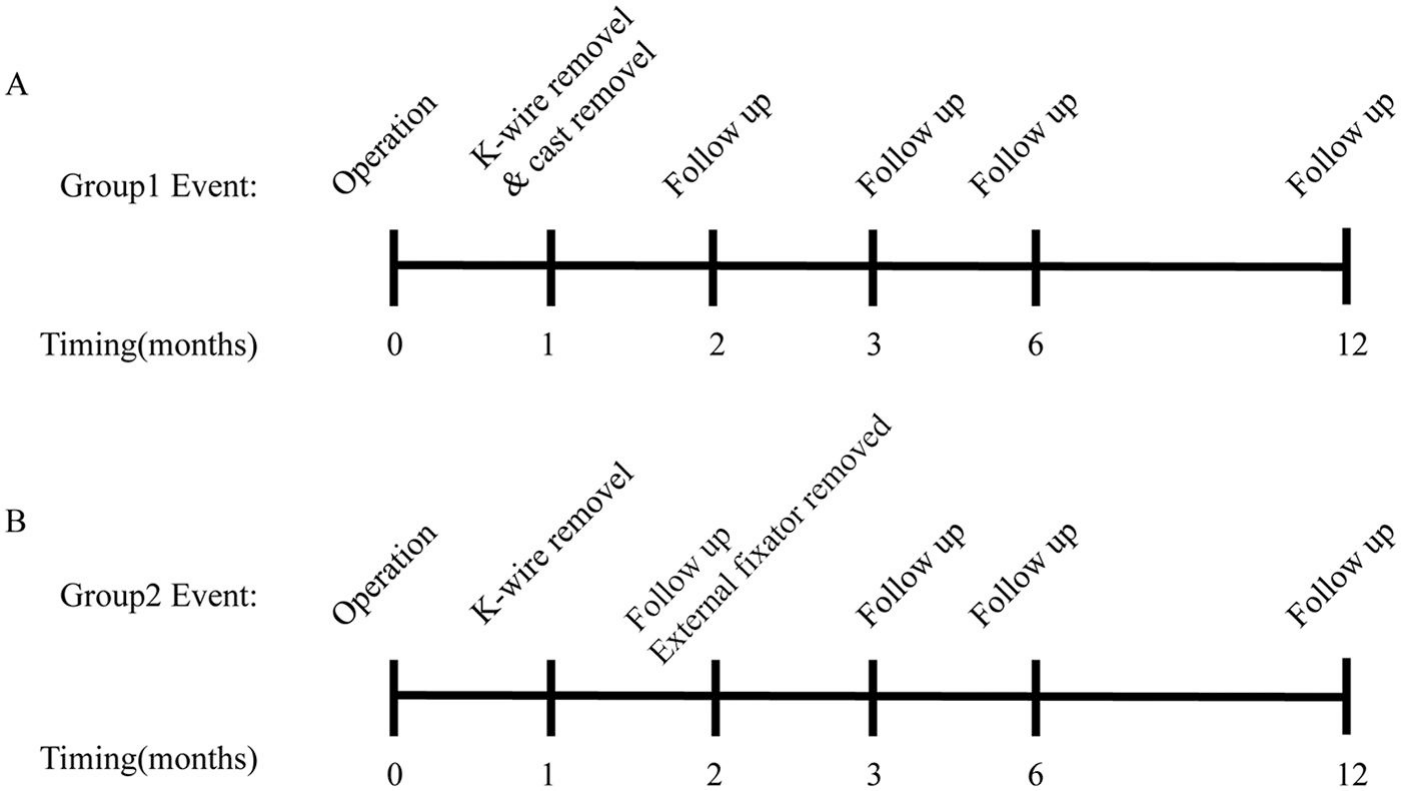
Post-operative care and follow-up. **(A)** Group 1 (K-wire fixation): The K-wires and cast were removed simultaneously 1 month after the operation. The follow-up timepoints are 1, 2, 3, 6, and 12 months post-surgery. **(B)** Group 2 (external fixation group): The K-wires were removed 1 month post-operatively and the external fixators were removed at 2 months.

Follow-up was scheduled at 1, 2, 3, 6, and 12 months post-operatively (Figure 2). Outcome measures included active elbow ROM (Figures 1I,J), Baumann angle (Figure 1F), humeral-capitellar angle (Figure 1G), carrying angle (Figure 1H), pin-tract infection, loss of reduction, and neurovascular status. Post-operative radiographs were obtained on post-operative day 1 and at each follow-up.

### Statistical analysis

Data were analyzed using SPSS 23.0. The normality of the continuous variables was tested using the Kolmogorov–Smirnov test. Age, gender, and side of injury are reported as numbers and percentages. Continuous variables (ROM, Baumann angle, carrying angle, and humeral-capitellar angle) are reported as medians and interquartile ranges (IQR). Between-group comparisons were performed using the Mann–Whitney *U* test. A *P*-value <0.05 was considered statistically significant.

## Result

A total of 52 patients were enrolled (37 in Group 1, 15 in Group 2). The mean age was 6.4 years (range 3–11 years) and 34 (65.4%) were male. In total, 35 patients (67.3%) had Gartland Type III fractures and 17 (32.7%) had Type II fractures. Finally, 31 (59.6%) had left-sided injuries.

### Baseline characteristics

Baseline demographics (age, gender, injured side, and fracture type) were comparable between the groups (all *P* > 0.05). The closed reduction rate was 64.9% in Group 1 and 86.7% in Group 2 (*P* = 0.042). No incidence of neurovascular deficit, vascular compromise, or internal fixation loosening occurred in either group. Two patients in Group 2 had mild pin-tract infections, which resolved after fixator removal and dressing changes (Table 1).

**Table 1:** Baseline demographic and clinical characteristics.

Parameter	Overall (*N* = 52)	Group 1, K-wire (*n* = 37)	Group 2, external fixation (*n* = 15)	*P*-value
Age, years, mean ± SD	6.4 ± 1.95	6.1 ± 1.8	7.0 ± 2.1	0.101
Male, *n* (%)	34 (65.4)	22 (59.5)	12 (80.0)	0.162
Right side, *n* (%)	21 (40.4)	15 (40.5)	6 (40.0)	0.972
Gartland Type III, *n* (%)	35 (67.3)	24 (64.9)	11 (73.3)	0.559
Closed reduction, *n* (%)	34 (65.4)	21 (56.8)	13 (86.7)	0.042*
Neurovascular deficit	0 (0)	0 (0)	0 (0)	–
Vascular compromise	0 (0)	0 (0)	0 (0)	–
Pin-tract infection	2 (3.8)	0 (0)	2 (13.3)	–
Fixation loosening	0 (0)	0 (0)	0 (0)	–

* *P* < 0.05 vs. Group 1.

### Radiological outcomes

The post-operative and 1-month Baumann and humeral-capitellar angles showed no significant between-group differences (all *P* > 0.05). At the 12-month follow-up, the median injured elbow flexion was 145° in Group 1 and 140° in Group 2; extension was 0° in both groups. The median ROM was 145° in both groups. Carrying angle and carrying angle loss were comparable between the groups (all *P* > 0.05) (Table 2).

**Table 2:** Radiological and functional outcomes at 12 months.

Parameter	Group 1 (*n* = 37)	Group 2 (*n* = 15)	*P-*value
Baumann's angle (post-op); median, IQR	75, 10	76, 9	0.626
Humeral-capitellar angle (post-op); median, IQR	37, 13	37, 7	0.714
Baumann's angle (1 month); median, IQR	73, 8	73, 8	0.503
Humeral-capitellar angle (1 month); median, IQR	39, 5	37, 10	0.496
Flexion (injured, 12 months); median, IQR	145, 10	140, 5	0.113
Extension (injured, 12 months); median, IQR	0, 2.5	0, 5	0.087
Flexion (contralateral); median, IQR	145, 5	145, 0	0.237
Extension (contralateral); median, IQR	0, 5	0, 5	0.964
ROM (injured, 12 months); median, IQR	145, 7.5	145, 5	0.653
ROM (contralateral); median, IQR	150, 5	150, 5	0.369
Carrying angle (injured, 12 months); median, IQR	10, 7.5	10, 10	0.638
Carrying angle (contralateral); median, IQR	10, 5	10, 5	0.964
Loss of carrying angle (12 months); median, IQR	0, 10	0, 5	0.649

*P* < 0.05 indicates a significant difference between Groups 1 and 2.

### Serial ROM assessment

**Figure 3:**
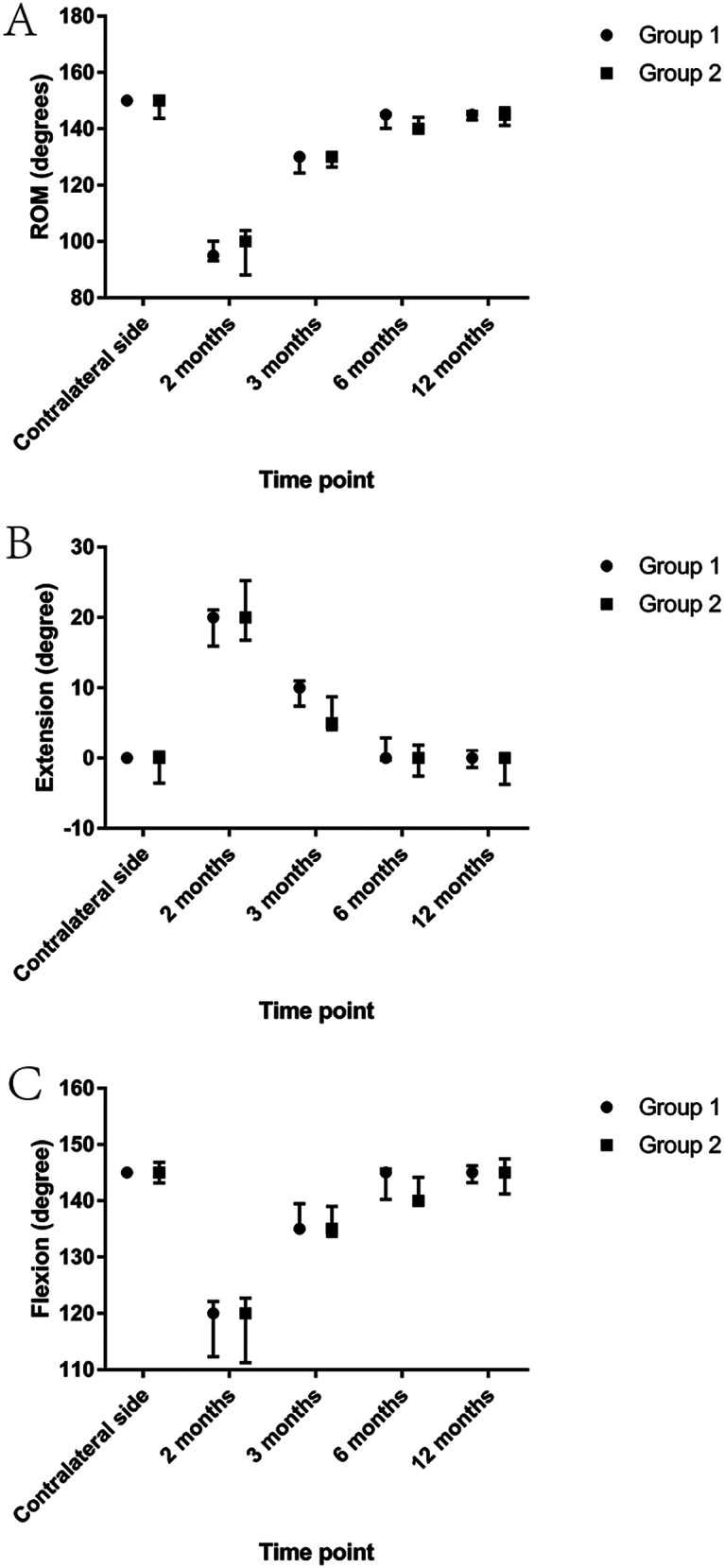
Comparison of elbow ROM **(A)**, extension **(B)**, and flexion **(C)** between the groups. (Median, error bars represent the interquartile range, 95% CI.)

Elbow flexion, extension, and ROM at 2, 3, 6, and 12 months showed no statistically significant differences between the groups (all *P* > 0.05) (Figure 3). Early restoration of elbow joint movement leads to a lower risk of joint stiffness (18). The children in Group 2 tended to delay active exercise due to discomfort until fixator removal at 2 months, offsetting the theoretical benefits of early mobilization.

### Flynn outcome grading

At final follow-up, all patients in both groups achieved excellent or good functional and cosmetic results per Flynn’s criteria (19). No fair or poor result was observed in either group (Table 3; statistical comparison: Fisher's exact test, *P* > 0.99).

**Table 3:** Evaluation of clinical outcome between two groups using Flynn's criteria.

Parameter	Group 1 (*n* = 37)	Group 2 (*n* = 15)
Functional: Loss of ROM		
Excellent (0–5)	30	13
Good (5–10)	7	2
Fair (10–15)	0	0
Poor (>15)	0	0
Cosmetic: Loss of carrying angle (degrees)		
Excellent (0–5)	37	15
Good (5–10)	0	0
Fair (10–15)	0	0
Poor (>15)	0	0

The data represent the number of patients in each group. Fisher's exact test, *p* > 0.99.

## Discussion

This retrospective study demonstrates that K-wire fixation and external fixation yield similarly excellent functional and radiological outcomes for high-level extension-type pediatric SHFs. This result is consistent with previous reports (13, 20–22). The only significant difference was a lower open reduction rate in the external fixation group, likely because Schanz pins serve as joysticks to facilitate closed reduction. The higher open reduction rate in the K-wire group may be reduced with an improved surgical technique and experience.

At the 12-month follow-up, elbow ROM and functional outcomes were comparable between the groups. No major complications (neurovascular injury, cubitus varus, and elbow stiffness) occurred in either group. Studies have suggested that external fixation may offer advantages over K-wire fixation, including a lower open reduction rate, greater rotational stability, a lower incidence of neurovascular complications, and earlier functional rehabilitation (13, 18, 23). Although external fixation theoretically allows earlier mobilization, children often avoid exercise until fixator removal at 2 months, negating this potential benefit. By contrast, K-wire fixation offers advantages including lower cost, simpler removal, lower infection risk, less scarring, and less soft-tissue injury.

This study has several limitations, including its single-center retrospective design with potential selection bias, a relatively small sample size (*n* = 52, with 15 patients in the external fixation group), unequal group sizes, and a follow-up period limited to 1 year, as longer follow-up is needed to assess late complications.

Alternative techniques, such as anterograde elastic intramedullary nails (ESINs), may be considered for high-level SHFs in select patients. Studies have suggested that ESINs offer potential advantages, including avoidance of iatrogenic ulnar nerve injury, earlier post-operative functional rehabilitation, and elimination of the need for cast immobilization (24). In our study, ESINs were not included in the comparison; therefore, our findings cannot be extrapolated to comparisons with ESINs. Future prospective randomized controlled trials or large-scale multicenter studies are warranted to systematically evaluate the indications, complications, and health economic benefits of these three techniques (external fixator, K-wires, and ESINs) for pediatric SHFs.

## Conclusion

External fixation is not superior to conventional K-wire fixation for high-level extension-type supracondylar humeral fractures in children. K-wire fixation is cost-effective, easy to remove, associated with lower infection risk and less soft-tissue trauma, and may be considered a preferred treatment option.

## Data Availability

The datasets presented in this study can be found in online repositories. The names of the repository/repositories and accession number(s) are as follows: DOI 10.5281/zenodo.18904598; https://zenodo.org/records/18904599.
