# Supplementary material for: External fixation is not superior to K-wire fixation in pediatric patients with high-level extension-type supracondylar humeral fractures
Source: Front Surg. 2026 Jul 15;13:1853760. doi: 10.3389/fsurg.2026.1853760 (PMC13412179; doi:10.3389/fsurg.2026.1853760)
Supplement: Supplementary file 1 [file Datasheet1.pdf]

Supplementary Material

Supplementary Figures

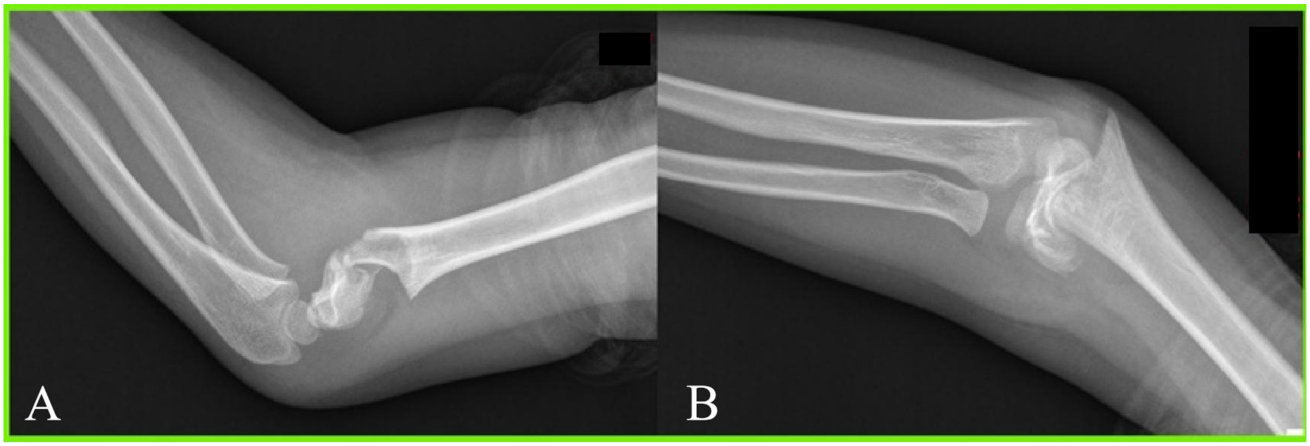

Supplementary Figure 1. Preoperative X-ray images of a Gartland Type Ⅲ supracondylar humeral fracture.
